# Chronic Urticaria—Pathogenesis, Diagnostics, Therapy and Influence of Coexisting Angioedema

**DOI:** 10.3390/jcm12020688

**Published:** 2023-01-15

**Authors:** Marzena Pluta-Kubicz, Zenon Brzoza

**Affiliations:** 1 Department of Internal Diseases, Allergology and Clinical Immunology, Faculty of Medical Sciences in Zabrze, Medical University of Silesia, 40-055 Katowice, Poland; 2 Department of Internal Diseases, Allergology, Endocrinology and Gastroenterology, Institute of Medical Sciences, University of Opole, 45-401 Opole, Poland

## 1. Urticaria—Types, Pathogenesis, Diagnostics and Therapy

Urticaria is one of the most frequent dermatological diseases and it usually occurs in paroxysmal, recurrent form. Urticarial wheal is a visible edema of the skin, which is caused by the increased permeability of blood vessels. Urticaria manifests as a sudden appearance of wheals, angioedema or both. Urticarial wheals usually disappear within 24 h, and angioedema in 72 h [1]. The most important role in the pathogenesis of this disease is played by histamine, which is released from mast cells. Immune mechanisms depend on IgE and its complement system. Non-immunological mechanisms, including the direct degranulation of mast cells, play a significant role as well. In about one third of patients diagnosed with chronic urticaria, IgG auto-antibodies to IgE or to IgE high-affinity receptors occur [2]. According to recent recommendations, urticaria has been divided into acute (AU), in which changes tend to resolve within 6 weeks, and chronic (CU), where skin changes occur for more than 6 weeks [1]. Chronic urticaria appears in spontaneous (CSU) and inducible (CIndU) forms—resulting from exposure to the causative agent. Among the inducible urticarias, we may distinguish: symptomatic dermographism—wheals appear 1–5 min after rubbing the skin; cold urticaria—evoked by cold air or water (approximately one third of physical urticarias); delayed pressure urticaria—provoked by pressure affecting the skin, painful edema usually appears after 3–12 h; solar urticaria—caused by ultraviolet or visible light; heat urticaria—contact with heat source; vibratory urticaria—evoked by vibration, i.e., jackhammer or vortex (very rare); cholinergic urticaria—wheals appear 2–15 min after physical activity or passive warming (not connected with anaphylaxis); contact urticaria—caused by latex, food (e.g., nuts, fish, shellfish), medicines (e.g., penicillin) or chemical substances (e.g., formaldehyde in clothing, resin, jellyfish, ammonium persulphate in cosmetics, food and clothing); and aquagenic urticaria—caused by contact with water at any temperature (extremely rare) [3].

Currently, two endotypes of CSU can be distinguished, which vary in terms of pathogenesis and markers of the disease, as well as clinical course and sensitivity to treatment applied: type I and IIb. Type I CSU is related to the occurrence of IgE antibodies to autoantigens such as TPO (thyroid peroxidase), TG (thyroglobulin), ds-DNA (double-stranded DNA) and Il-24. In type I, it is observed that the occurrence of allergic diseases and the total concentration of IgE are normal or even increased. Antihistamines and omalizumab used in this endotype therapy are usually effective [4]. In type IIb CSU, IgG and IgM antibodies to IgE and high-affinity receptors for IgE (FceRI) are identified. Additionally, in the clinical course of this endotype, greater severity of symptoms and a prolonged duration of the disease, coexistence of autoimmune diseases, presence of ANA (antinuclear antibodies), elevated CRP levels, basopenia and eosinopenia in peripheral blood and reduced levels of total IgE occur. During the treatment, lower response to antihistamines and omalizumab is observed, however, immunosuppressive therapy is usually effective [4].

The diagnosis of urticaria is based on the skin lesions’ appearance, and its causes are potentially determined through carefully collected medical history and diagnostic tests. The following diagnostic procedures are recommended for urticaria: basic laboratory tests (peripheral blood morphology, ESR, CRP, tIgE, anti-TPO, anti-Tg), as well as additional tests in more complex cases, such as ANA, cryoglobulins, components of the complement system, number of eosinophils in peripheral blood, skin prick tests (SPT), autologous serum skin tests, patch tests, provocation tests with food and medications, skin biopsy (in cases of suspected urticarial vasculitis, mastocytosis or treatment-resistant CSU) and physical tests—exercise, warm bath provocation, temperature and pressure [5].

In the treatment of urticaria, the elimination of the triggering factors and avoiding factors that intensify the symptoms play a major role. The basics of first-line symptomatic treatment are second generation antihistamines (AH). In case of continuous disease with a lack of control after applying the standard dosage of AH, it is suggested to increase the dosage four-fold. The third line of therapy is adding the AH human monoclonal antibody—omalizumab. Nowadays, biological treatment with omalizumab is a recommended therapy for patients over the age of twelve. The medication is administered subcutaneously in 2–4-week intervals. [6,7]. The fourth line of therapy involves treatment with the additional use of ciclosporin instead of omalizumab [8]. In case of symptom exacerbation while using any of the strategies mentioned, the short application of systemic glucocorticoids (maximum 10 days) is allowed.

## 2. Angioedema as a Form of Urticaria Manifestation

Angioedema (AE) is an edema of the subcutaneous or submucosal tissue, which develops as a result of an extension and increase in vascular permeability. Most frequently, this symptom is well demarcated, asymmetrical, located around the eyelids, lips, genital areas and distal parts of the limbs, as well as the mucous membrane of the upper respiratory tract. It usually occurs in the form of a single focus rather than several changes of different sizes. Skin lesions may be painful. Recurrent edema might appear in the same location. Laryngeal and/or throat edema with dyspnea is life-threatening and potentially related to acute respiratory failure caused by the obstruction of the upper respiratory tract [9]. AE frequently coexists with wheals and resolves within 24–72 h. The frequency of its occurrence is estimated at approximately 1:10,000–1:150,000 [10,11]. Stress may play a significant role in the occurrence and/or exacerbation of the disease [12]. A crucial part of the diagnostics of recurrent angioedema is collecting the patient’s detailed medical history, determining whether it was accompanied by urticarial wheals, what medication was administered before the edema occurred, what did the patient eat, whether the edema runs in the family and if the patient encountered stressful situations that may have led to the angioedema. In the event of a life-threatening angioedema, it is essential to administer epinephrine by intramuscular injection [13,14].

## 3. The Impact of Angioedema and Wheals on Quality of Life

A significant increase in the interest in diseases affecting patients’ quality of life has been observed in recent years. It does not only refer to urticaria and its angioedema form, but also the deterioration in quality of life caused by the coexistence of these symptoms. Undoubtedly, the symptoms of CSU are a cause of distress for patients, e.g., itching intensity in CSU has been proven to be related to stress [4,5]. One of the first studies assessing urticaria patients’ quality of life was performed by O’Donnell et al. [15]. This research was revolutionary, as the authors showed that the deterioration in quality of life during a non-life-threatening disease such as urticaria is comparable with the decrease in quality of life in case of serious heart disease. Reports by other authors have also confirmed lower quality of life in patients with urticaria when compared to patients with allergic diseases of the respiratory tract [16]. About 50% of patients with angioedema also manifest wheals, and 40% of urticaria patients demonstrate wheals only. Isolated angioedema appears in 10–15 % of urticaria patients [17]. Until recently, not much scientific research had been devoted specifically to angioedema. Coexisting angioedema was found to be a risk factor of more severe urticaria course and symptom reoccurrence in a 5-year follow-up [12]. In general, an additional effect of angioedema coexisting with wheals on different aspects of the disease course seems to be significant, though relatively poorly explored and measured. We know that similar clinical symptoms, such as edema attacks in the course of hereditary angioedema, can provoke symptoms of depression and anxiety [18]. The negative influence of CSU symptoms on patients’ emotions and quality of life is undoubtable [19]. Clinical experience proves angioedema symptoms in the course of CSU, especially in a specific location, to result in emotional disturbances. We do not know if CSU symptoms, wheals or angioedema cause a similar effect. One third of patients with chronic urticaria have symptoms that are induced by chemical or physical factors, and avoiding them causes a significant limitation in their quality of life [17]. Angioedema may be disfiguring and painful. Situated around the hands, feet, and joints, it limits patients’ functioning and, as a consequence, their daily activities and professional life. What is more, episodes of face edema and oral cavity edema cause difficulties in breathing, and, therefore, cause patients to be frightened of suffocation. The constant feeling of fear may lead to sleep disturbances, and is a frequent cause of visits to emergency units [20]. Angioedema is proven to result in patients’ anxiety development, but the underlying mechanisms and ways of identification of patients who are particularly susceptible to manifesting emotional disturbances have not been thoroughly explored [21]. Until recently, not much scientific research has been focused on angioedema as a form coexisting with urticaria wheals. Persistent pruritus in chronic urticaria and the fear of angioedema attack are the main causes of sleep disorders, which, consequently, lead to emotional disturbances and the deterioration of physical wellbeing during the day [22]. These symptoms also have an impact on patients’ partners, constituting a potential basis for sexual dysfunctions [23]. Patients’ social life, ability to perform physical activity and effectiveness in a professional capacity become affected [22]. In previous studies, it has been put forward that the emotional state, especially the anxiety, of patients with angioedema contributes to a significant decrease in their quality of life [21]. In this group, there is a lowered sense of coherence, which contributes to an increased manifestation of anxiety [24]. Identifying patients with symptoms of angioedema and a lowered sense of coherence may contribute to improving the effectiveness of therapy in this group [24].

## 4. Conclusions

Urticaria is a multiform disorder with partially unknown pathophysiology, and therapy effectiveness is still limited, in spite of several options. Recurrent angioedema is a form of chronic urticaria. The symptoms of angioedema, with or without coexisting wheals, constitute a significant cause of the deterioration in urticaria patients’ quality of life. A lower sense of coherence in this group of patients leads to an increased manifestation of anxiety. Identifying patients with angioedema symptoms and a lowered sense of coherence may potentially create wider interdisciplinary therapeutic possibilities for this group.
